# Cardiovascular disease risk prediction using automated machine learning: A prospective study of 423,604 UK Biobank participants

**DOI:** 10.1371/journal.pone.0213653

**Published:** 2019-05-15

**Authors:** Ahmed M. Alaa, Thomas Bolton, Emanuele Di Angelantonio, James H. F. Rudd, Mihaela van der Schaar

**Affiliations:** 1 University of California Los Angeles, Los Angeles, California, United States of America; 2 Department of Public Health and Primary Care, University of Cambridge, Cambridge, United Kingdom; 3 National Institute for Health Research (NIHR) Blood and Transplant Research Unit (BTRU) in Donor Health and Genomics, University of Cambridge, Cambridge, United Kingdom; 4 Department of Cardiovascular Medicine, University of Cambridge and Cambridge University Hospitals NHS Foundation Trust, Cambridge, United Kingdom; 5 University of Oxford, Oxford, United Kingdom; 6 Alan Turing Institute, London, United Kingdom; University of Tampere, FINLAND

## Abstract

**Background:**

Identifying people at risk of cardiovascular diseases (CVD) is a cornerstone of preventative cardiology. Risk prediction models currently recommended by clinical guidelines are typically based on a limited number of predictors with sub-optimal performance across all patient groups. Data-driven techniques based on machine learning (ML) might improve the performance of risk predictions by agnostically discovering novel risk predictors and learning the complex interactions between them. We tested (1) whether ML techniques based on a state-of-the-art automated ML framework (AutoPrognosis) could improve CVD risk prediction compared to traditional approaches, and (2) whether considering non-traditional variables could increase the accuracy of CVD risk predictions.

**Methods and findings:**

Using data on 423,604 participants without CVD at baseline in UK Biobank, we developed a ML-based model for predicting CVD risk based on 473 available variables. Our ML-based model was derived using *AutoPrognosis*, an algorithmic tool that automatically selects and tunes ensembles of ML modeling pipelines (comprising data imputation, feature processing, classification and calibration algorithms). We compared our model with a well-established risk prediction algorithm based on conventional CVD risk factors (Framingham score), a Cox proportional hazards (PH) model based on familiar risk factors (i.e, age, gender, smoking status, systolic blood pressure, history of diabetes, reception of treatments for hypertension and body mass index), and a Cox PH model based on all of the 473 available variables. Predictive performances were assessed using area under the receiver operating characteristic curve (AUC-ROC). Overall, our AutoPrognosis model improved risk prediction (AUC-ROC: 0.774, 95% CI: 0.768-0.780) compared to Framingham score (AUC-ROC: 0.724, 95% CI: 0.720-0.728, *p* < 0.001), Cox PH model with conventional risk factors (AUC-ROC: 0.734, 95% CI: 0.729-0.739, *p* < 0.001), and Cox PH model with all UK Biobank variables (AUC-ROC: 0.758, 95% CI: 0.753-0.763, *p* < 0.001). Out of 4,801 CVD cases recorded within 5 years of baseline, AutoPrognosis was able to correctly predict 368 more cases compared to the Framingham score. Our AutoPrognosis model included predictors that are not usually considered in existing risk prediction models, such as the individuals’ usual walking pace and their self-reported overall health rating. Furthermore, our model improved risk prediction in potentially relevant sub-populations, such as in individuals with history of diabetes. We also highlight the relative benefits accrued from including more information into a predictive model (information gain) as compared to the benefits of using more complex models (modeling gain).

**Conclusions:**

Our AutoPrognosis model improves the accuracy of CVD risk prediction in the UK Biobank population. This approach performs well in traditionally poorly served patient subgroups. Additionally, AutoPrognosis uncovered novel predictors for CVD disease that may now be tested in prospective studies. We found that the “information gain” achieved by considering more risk factors in the predictive model was significantly higher than the “modeling gain” achieved by adopting complex predictive models.

## Introduction

Globally, cardiovascular disease (CVD) remains the leading cause of morbidity and mortality [1]. Current clinical guidelines for primary prevention of CVD emphasize the need to identify asymptomatic patients who may benefit from preventive action (e.g., initiation of statin therapy [2]) based on their predicted risk [3–6]. Different guidelines recommend different algorithms for risk prediction. For example, the 2010 American College of Cardiology/American Heart Association (ACC/AHA) guideline [7] recommended use of Framingham Risk Score [4], whereas the 2016 European guidelines recommended use of the Systematic Coronary Risk Evaluation (SCORE) algorithm [8]. In the UK, the current National Institute for Health and Care Excellence (NICE) guidelines recommend use of the QRISK2 score to guide the initiation of lipid lowering therapies [9, 10].

Existing risk prediction algorithms are typically developed using multivariate regression models that combine information on a limited number of well-established risk factors, and generally assume that all such factors are related to the CVD outcomes in a linear fashion, with limited or no interactions between the different factors. Because of their restrictive modeling assumptions and limited number of predictors, existing algorithms generally exhibit modest predictive performance [11], especially for certain sub-populations such as individuals with diabetes [12–15] or rheumatoid arthritis [3]. Data-driven techniques based on machine learning (ML) can improve the performance of risk predictions by exploiting large data repositories to agnostically identify novel risk predictors and more complex interactions between them. However, only a few studies have investigated the potential advantages of using ML approaches for CVD risk prediction, focusing only on a limited number of ML methods [16, 17] or a limited number of risk predictors [18].

Here, we aim to assess the potential value of using ML approaches to derive risk prediction models for CVD. We analyzed data on 423,604 participants without CVD at baseline in UK Biobank, a large prospective cohort study in which participants were recruited from 22 centers throughout the UK. We used a state-of-the-art automated ML method (AutoPrognosis) to develop ML-based risk prediction models and evaluated their predictive performances in the overall population and clinically relevant sub-populations. In this paper, we do not focus on the algorithmic aspects of the ML methods involved and rather focus on their clinical application. Methodological details on our automated ML algorithm can be found in our technical publication in [19].

## Materials and methods

### Study design and participants

Participants were enrolled in the UK Biobank from 22 assessment centers across England, Wales, and Scotland, during the period spanning from 2006 to 2010 [20]. We extracted a cohort of participants who were 40 years of age or older and had no known history of CVD at baseline. That is, patients with previous history of coronary heart disease, other heart disease, stroke, transient ischaemic attack, peripheral arterial disease, or cardiovascular surgery were excluded from the analysis. The total number of participants who met the inclusion criteria was 423,604. The last available date of participant follow-up was Feb 17, 2016. UK Biobank obtained approval from the North West Multi-centre Research Ethics Committee (MREC), and the Community Health Index Advisory Group (CHIAG). All participants provided written informed consent prior to enrollment in the study. The UK Biobank protocol is available online [21].

The UK Biobank dataset keeps track of a large number of variables for each participant, but most of those variables are missing for most patients. In order to include the maximum possible number of (informative) variables in our analysis, we included all variables that are missing for less than 50% of patients with CVD outcomes. This corresponded to a rate of missingness of 85% for the entire population of participants. Our rationale for assessing the missingness rate among patients with CVD is that missingness itself maybe informative (i.e., the chance of a variable being missing may depend on the outcome). By excluding all variables that were missing for more than 85% of the participants, a total of 473 variables were included in our analysis. We categorized all variables in the UK Biobank into 9 categories: health and medical history, lifestyle and environment, blood assays, physical activity, family history, physical measures, psychosocial factors, dietary and nutritional information, and sociodemographics [22]. The (categorized) lists of variables involved in our analysis are provided in the supporting information (S1 to S9 Tables).

### Outcome

The primary outcome was the first fatal or non-fatal CVD event. A CVD event was defined as the assignment of any of the ICD-10 diagnosis codes F01 (vascular dementia), I20-I25 (coronary/ischaemic heart diseases), I50 (heart failure events, including acute and chronic systolic heart failures), and I60-I69 (cerebrovascular diseases), or any of the ICD-9 codes 410-414 (ischemic heart disease), 430-434, and 436-438 (cerebrovascular disease). Follow-up data was obtained from the hospital episode statistics (a data warehouse containing records of all patients admitted to NHS hospitals), and the equivalent datasets in Scotland and Wales [23].

### Models tested

#### Framingham Risk Score

At the time of conducting this study, the UK Biobank had not yet released data on the participants’ total cholesterol, HDL cholesterol and LDL cholesterol, which are used as predictors in various established algorithms, such as Framingham score [4], ACC/AHA [24], QRISK2 [9], and SCORE [5]. The Framingham score, however, provides an incarnation of its underlying model based on nonlaboratory predictors, which replaces lipids with Body Mass Index (BMI) [4]. Since BMI is currently collected for 99.38% of the UK Biobank participants, we compared our model with the BMI version of the Framingham score. We used the published predicting equations (beta-coefficients and survival functions) of the BMI-based Framingham model developed in [4]. (Framingham risk calculator and model coefficients are publicly available in: https://www.framinghamheartstudy.org.)

The Framingham score is based on 7 core risk factors: gender, age, systolic blood pressure, treatment for hypertension, smoking status, history of diabetes, and BMI. All of those variables were complete for the participants in the extracted cohort, with the exception of systolic blood pressure (missing for 6.8% of the participants), and BMI (missing for 0.62% of the participants). We used the *MissForest* non-parametric data imputation algorithm [25] to recover the missing values. Using the MissForest algorithm, we sampled 5 imputed datasets and averaged the model predictions for each participant on the 5 datasets (this is known in the literature as Rubin’s rules [25]). The number of imputed datasets was selected via cross-validation.

#### Cox proportional hazards model

We evaluated the performance of two Cox Proportional Hazards (PH) models derived from the analysis cohort: a model that only uses the traditional 7 risk factors used by the Framingham score, and a model that uses all of the 473 variables in the UK Biobank. To fit the Cox PH models, we imputed the missing data using the MissForest imputation algorithm (with 5 imputations). The Cox PH model that uses the traditional 7 risk factors used by Framingham score can be thought of as a variant of Framingham score calibrated to the UK population (the Framingham score was originally derived for a US population). For the Cox PH model that uses all of the 473 predictors, we applied variable selection using the LASSO method [26]. (Variable selection was applied since fitting the Cox model with all variables resulted in an inferior performance due to the numerical collapse of the Cox model solvers in high dimensions.) To apply variable selection, we fit a LASSO regression model (a linear model penalized with the L1 norm) to predict the (binary) CVD outcomes. The fitted model gives a sparse solution whereby many of the estimated coefficients are zero. We select all the variables with non-zero coefficients in the fitted LASSO model and feed those variables into a Cox model fitted on the same batch of data. We optimize the LASSO model regularization parameter via cross-validation.

#### Standard ML models

We considered 5 standard ML benchmarks that cover different classes of ML modeling approaches. The models under consideration are: linear support vector machines (SVM) [27] (a linear classifier), random forest [28] (a tree-based ensemble method), neural networks [29] (a deep learning method), AdaBoost [30] and gradient boosting machines [31] (boosting ensemble methods). (We also attempted to fit a kernel SVM, but fitting such a model was computationally infeasible for the UK Biobank cohort because it entails a cubic complexity in the number of datapoints.) The purpose of including those models in our experimental evaluations is to ensure that AutoPrognosis has automatically selected and tuned the best possible ML model, and that no individually-tuned ML model performed better than the model selected by AutoPrognosis. (We decided to include a Gradient boosting model in retrospect because it was assigned the largest weight in the ensemble formed by AutoPrognosis.) We implemented all these models using the Scikit-learn library in Python programming language [32]. The models’ hyper-parameters were determined via grid search. Data imputation for all models was conducted using the MissForest algorithm (with 5 imputed datasets). (We have attempted other imputation algorithms, such as multiple imputation by chained equations, but MissForest provided a better predictive performance.)

### Model development using AutoPrognosis

**Fig 1:**
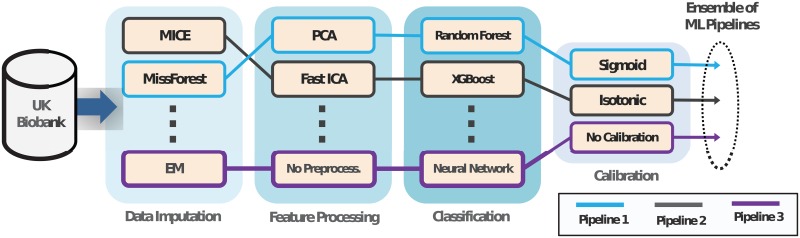
An illustrative schematic for AutoPrognosis. In this depiction, AutoPrognosis constructs an ensemble of three ML pipelines. Pipeline 1 uses the MissForest algorithm to impute missing data, and then compresses the data into a lower-dimensional space using the principal component analysis (PCA) algorithm, before using the random forest algorithm to issue predictions. Pipelines 2 and 3 use different algorithms for imputation, feature processing, classification and calibration. AutoPrognosis uses the algorithm in [19] to make decisions on what pipelines to select and how to tune the pipelines’ parameters.

We developed an ML-based model for CVD risk prediction using AutoPrognosis, an algorithmic framework for automating the design of ML-based clinical prognostic models [19]. A schematic for the AutoPrognosis framework is provided in Fig 1. Given the participants’ variables and CVD outcomes, AutoPrognosis uses an advanced Bayesian optimization technique [33, 34] in order to (automatically) design a prognostic model made out of a weighted ensemble of ML pipelines. Each ML pipeline comprises design choices for data imputation, feature processing, classification and calibration algorithms (and their hyper-parameters). (Calibration means that the numerical outputs of a model correspond to the actual risk of a CVD event. That is, an output prediction of 20% means that the patient’s 5-year risk of a CVD event is 20%.) The design space of AutoPrognosis contains 5,460 possible ML pipelines (7 possible imputation algorithms, 9 feature processing algorithms, 20 classification algorithms, and 3 calibration methods). The list of algorithms that constitute the design space of AutoPrognosis is provided in Table 1. A detailed technical and methodological description of AutoPrognosis can be found in our previous work in [19].

**Table 1:** List of algorithms included in AutoPrognosis.

Pipeline Stage	Algorithms		
**Data Imputation**	missForest Mean MICE	Median EM None	Most-frequent Matrix completion
**Feature Process**	Feature agglomeration R. kitchen sinks Select Rates	Kernel PCA Fast ICA Nystroem	Polynomial PCA Linear SVM
**Classification**	Bernoulli NB Linear SVM Gaussian NB Multinomial NB Light GBM Survival Forest Cox Regression	AdaBoost Gradient Boosting XGBoost Random Forest Logistic Regression Bagging Ridge Classifier	Decision Tree LDA Extr. Random Trees Neural Network Gaussian Process *k*-NN
**Calibration**	Calibration	Sigmoid	none

MICE: multiple imputation by chained equations, EM: expectation maximization, PCA: principal component analysis, ICA: independent component analysis, SVM: support vector machines, NB: Naïve Bayes, NN: nearest neighbors, LDA: linear discriminant analysis, GBM: gradient boosting machine.

To train our model, we set AutoPrognosis to conduct 200 iterations of the Bayesian optimization procedure in [19], where in each iteration the algorithm explores a new ML pipeline and tunes its hyper-parameters. Cross-validation was used in every iteration to evaluate the performance of the pipeline under evaluation. The (in-sample) model learned by AutoPrognosis combined 200 weighted ML pipelines, the strongest of which comprised the MissForest data imputation algorithm, no feature processing steps, an *XGBoost* ensemble classifier (with 200 estimators) [35], and sigmoid regression for calibration. Details of the model learned by AutoPrognosis is provided in the supporting information (S1 Appendix). In the Results Section, we will directly refer to our model as “AutoPrognosis”.

### Variable ranking

In order to identify the relative importance of the 473 variables used to build our model, we use a *post-hoc* approach to rank the contribution of the different variables in the predictions issued by the model. The ranking is obtained by fitting a random forest model with the participants’ variables as the inputs, and the predictions of our model as the outputs, and then assigning variable importance scores to the different variables using the standard permutation method in [36]. Using the permutation method, we assess the mean decrease in classification accuracy for every variable after permuting that variable over all trees. The resulting variable importance scores reflect the impact each variable has on the predictions issued by AutoPrognosis. We used the random forest algorithm for post-hoc variable ranking because it is a nonparametric algorithm that can recognize complex patterns of variable interaction while enabling principled evaluation of variable importance [36]. Other variable ranking methods based on associative classifiers (such as the one proposed in [19]) entail a computational complexity that is exponential in the number of variables, and hence are not suitable for our study as it involves more than 400 variables.

To disentangle the “modeling gain” achieved by utilizing ML-based techniques from the “information gain” achieved by just using more variables, we created a simpler version of AutoPrognosis that only uses the same 7 core risk factors (age, gender, systolic blood pressure, smoking status, treatment of hypertension, history of diabetes, and BMI) used by the existing prediction algorithms. In addition, we created another version of the AutoPrognosis model that uses only non-laboratory variables in UK Biobank.

### Statistical analysis

In order to avoid over-fitting, we evaluated the prediction accuracy of all models under consideration via 10-fold stratified cross-validation using area under the receiver operating characteristic curve (AUC-ROC). In every cross-validation fold, a training sample (381,244 participants) was used to derive the Cox PH models, standard ML models, and our model (AutoPrognosis), and then a held-out sample (42,360 participants) was used for performance evaluation. We report the mean AUC-ROC and the 95% confidence intervals (Wilson score intervals) for all models. The calibration performance of our model was evaluated via the Brier score.

## Results

### Characteristics of the study population

A total of 423,604 participants had sufficient information for inclusion in this analysis. Overall, the mean (SD) age of participants at baseline was 56.4 (8.1) years, and 188,577 participants (44.5%) were male. Over a median follow-up of 7 years (5th-95th percentile: 5.7-8.4 years; 3 million person-years at risk), there were 6,703 CVD cases. The mean age of CVD cases was 60.5 years (60.2 years for men and 61.1 years for women). Because the minimum follow-up period for all participants was 5 years, we evaluated the accuracy of the different models in predicting the 5-year risk of CVD. At a 5-year horizon, the total number of CVD cases was 4,801.

### Prediction accuracy

#### Comparison of prediction models

The prediction accuracy of the different models under consideration evaluated at a 5-year horizon is shown in Table 2. We used the Framingham score as a baseline model for performance evaluation (AUC-ROC: 0.724, 95% CI: 0.720-0.728). Both the Cox PH model with the 7 conventional risk factors (AUC-ROC: 0.734, 95% CI: 0.729-0.739), and the Cox PH model with all variables (AUC-ROC: 0.758, 95% CI: 0.753-0.763) achieved an improvement in the AUC-ROC compared to the baseline model (*p* < 0.001). The improvement achieved by the Cox PH model that uses the same predictors used by the Framingham score is due in part to the fact that the Cox PH model is directly derived from the analysis cohort, whereas the Framingham score coefficients were derived from a different population.

**Table 2:** Performance of all prediction models under consideration.

Model	AUC-ROC	Absolute AUC-ROC Change
Framingham Score	0.724 ± 0.004	Baseline model
Cox PH Model (7 core variables)	0.734 ± 0.005	+ 1.0%
Cox PH Model (all variables)	0.758 ± 0.005	+ 3.4%
Support Vector Machines	0.709 ± 0.061	- 1.5%
Random Forest	0.730 ± 0.004	+ 0.6%
Neural Networks	0.755 ± 0.005	+ 3.1%
AdaBoost	0.759 ± 0.004	+ 3.5%
Gradient Boosting	0.769 ± 0.005	+ 4.5%
AutoPrognosis (7 core variables)	0.744 ± 0.005	+ 2.0%
AutoPrognosis (369 non-lab. variables)	0.761 ± 0.005	+ 3.7%
AutoPrognosis (104 lab. variables)	0.735 ± 0.008	+ 1.1%
AutoPrognosis (all variables)	0.774 ± 0.005	+ 5.0%

The Framingham score is provided as the reference model for comparative purposes.

With the exception of support vector machines, all the standard ML models achieved statistically significant improvements compared to the baseline Framingham score. Furthermore, when compared to the Cox PH model that uses all variables, neural networks, AdaBoost, gradient boosting, and AutoPrognosis all achieved a significantly higher AUC-ROC. AutoPrognosis achieved a higher AUC-ROC compared to all other standard ML models (AUC-ROC: 0.774, 95% CI: 0.768-0.780, *p* < 0.001), which suggests that the automated ML system managed to automatically select and tune the “right” ML model. (The AutoPrognosis model trained on all variables was also well-calibrated, with an in-sample Brier score of 0.0121.) Compared to the most competitive benchmark (the Cox PH model that uses all of the variables), the net re-classification index (NRI) was +12.5% in favor of AutoPrognosis. AutoPrognosis trained only with the 7 conventional risk factors still outperformed the baseline Framingham score (*p* < 0.001).

Most of the variables in the UK Biobank are non-laboratory variables collected through an automated touchscreen questionnaire about lifestyle, clinical history and nutritional habits. We evaluated the accuracy of AutoPrognosis once when it is trained with 369 variables corresponding to the participants’ self-reported information (questionnaires) only, and once when it is trained with 104 variables obtained from blood assays, diagnostic tests, and physiological measurements. As we can see in Table 2, AutoPrognosis with only questionnaire-related variables still achieves a significant improvement over the baseline Framingham score (AUC-ROC: 0.752, 95% CI: 0.747-0.757, *p* < 0.001), and is superior to the model that only uses laboratory-based variables.

#### Classification analysis

In order to better assess the clinical significance of our results, we compared the AutoPrognosis model with the traditional Framingham score in predicting 7.5% CVD risk (threshold for initiating lipid-lowering therapies recommended by the NICE guidelines [10]). At this operating point, the Framingham baseline model predicted 2,989 CVD cases correctly from 4,801 total cases, resulting in a sensitivity of 62.2% and PPV of 1.5%. Our AutoPrognosis model correctly predicted 3,357 out of the 4,801 CVD cases, resulting in a sensitivity of 69.9% and PPV of 2.6%. This corresponds to 368 net increase in the number of CVD patients who would benefit from receiving a preventive treatment in a timely manner when utilizing the predictions of our model.

#### Variable importance

Table 3 lists the 20 most important variables ranked according to their contribution to the predictions of the AutoPrognosis model (along with their importance scores). Variables related to physical activity (usual walking pace) and information on blood measurements appeared to be more important for the predictions of AutoPrognosis than traditional risk factors included in most existing scoring systems. For women, a remarkable predictor of CVD risk was the measured “ankle spacing width”. This may be linked to symptoms of poor circulation, such as swollen legs, which is predictive of future CVD events [37]. We also found that usage of hormone-replacement therapy (HRT) was on the list of top predictors of CVD risk for women. For men, blood measurements such as haematocrit percentage and haemoglobin concentration, and variables such as urinary sodium concentration were among the most important risk factors.

**Table 3:** Variable ranking by their contribution to the predictions of AutoPrognosis.

Variable (Men)	Score	Variable (Women)	Score
Age*	0.346	Age*	0.370
Smoking*	0.101	Smoking*	0.099
Usual walking pace	0.052	Usual walking pace	0.057
Systolic blood pressure*	0.040	Ankle spacing width	0.035
Microalbumin in urine	0.032	Self-reported health rating	0.030
High blood pressure	0.030	Systolic blood pressure*	0.026
Red blood cell distribution width	0.025	High blood pressure	0.024
Self-reported health rating	0.019	Red blood cell distribution width	0.023
Haematocrit percentage	0.014	Microalbumin in urine	0.017
Father age at death	0.014	Father age at death	0.017
BMI*	0.013	White blood cell count	0.011
Diastolic blood pressure	0.012	Number of Treatments	0.011
White blood cell count	0.012	Mean reticulocyte volume	0.008
Impedance of arm (left)	0.009	Leg predicted mass (right)	0.006
Haemoglobin concentration	0.007	Neutrophill count	0.006
Neutrophill count	0.005	Basal metabolic rate	0.005
Number of Treatments	0.004	Hormone-replac. therapy usage	0.005
Mean reticulocyte volume	0.004	Blood clot in the leg	0.004
Urinary sodium concentration	0.004	Forced expiratory volume	0.004
Monocyte count	0.004	Duration of fitness test	0.004

* Risk factors utilized by existing risk prediction algorithms.

Explanations for the different variables in this table are provided in S2 Appendix.

#### Prediction accuracy in individuals with history of diabetes

Among the 423,604 participants included in our cohort, a total of 17,908 participants (4.22%) had a known history of diabetes (either Type 1 or Type 2) at baseline. In Table 4, we show the AUC-ROC performance of AutoPrognosis and the baseline Framingham score when validated separately on the diabetic and non-diabetic populations. As we can see, the baseline Framingham score was less accurate in the diabetic population (AUC-ROC: 0.578, 95% CI: 0.560-0.596) compared to its achieved accuracy for the overall population (AUC-ROC: 0.724, 95% CI: 0.720-0.728, *p* < 0.001). On the contrary, AutoPrognosis maintained high predictive accuracy for the diabetic population (AUC-ROC: 0.713, 95% CI: 0.703-0.723).

**Table 4:** Performance of AutoPrognosis in the diabetic patient subgroup.

Model	AUC-ROC (No diabetes)	AUC-ROC (Diabetes)
Framingham score	0.724 ± 0.004	0.578 ± 0.018
AutoPrognosis	0.774 ± 0.005	0.713 ± 0.010

Performance of AutoPrognosis and the Framingham score validated separately on a testing cohort of diabetic patients (1,790 participants), and a testing cohort of non-diabetic patients (40,570 participants) via 10-fold cross-validation. AutoPrognosis was trained using the entire training cohort that combines both diabetic and non-diabetic individuals (381,244 participants).

The variable ranking for the diabetic sub-population is provided in Table 5. We note that the list of important variables in the diabetic subgroup is substantially different from that of the overall population. One major difference is that for diabetic patients, microalbuminuria appeared to be strongly linked to an elevated CVD risk. In the overall population (423,604 participants), the average measure of microalbumin in urine was 27.8 mg/L for participants with no CVD events, and 52.2 mg/L for participants with CVD events. In the diabetic population (17,908 participants), participants with no CVD events had an average microalbumin in urine of 61.0 mg/L, whereas for those with a CVD event, the average microalbumin in urine was 128.76 mg/L. (Information on microalbumin in urine was available for 30% of the patients in the overall population, and 50% of patients in the diabetic population.)

**Table 5:** Variable ranking for the diabetic population.

Variable	Score
Age	0.207
**Microalbumin in urine**	0.110
Usual walking pace	0.078
Smoking status	0.064
Systolic blood pressure	0.034
Red blood cell distribution width	0.027
Neutrophill count	0.018
Number of Treatments	0.018
High blood pressure	0.014
Urinary sodium concentration	0.014

#### Predictive ability of individual variables in UK Biobank

**Fig 2:**
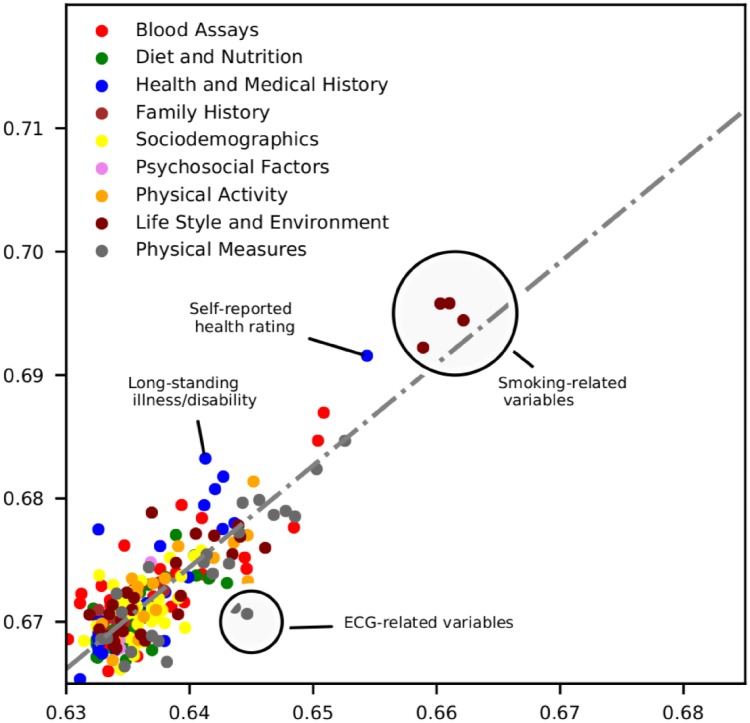
Predictive ability of the UK Biobank variables for men and women. Each point represents a variable in the UK Biobank ordered by the ability to predict CVD events for men and women. Predictions based solely on age achieved an AUC-ROC of 0.632 ± 0.003 for men and 0.665 ± 0.002 for women. We report the AUC-ROC from models trained with individual variables in addition to age, and only display variables that achieved a statistically significant improvement in AUC-ROC compared to predictions based on age only. Each color represents a different variable category. Variables deviating from the (dotted gray) regression line have an AUC-ROC that differs between men and women more than expected in view of the overall association between the two genders, suggesting a stronger relative importance in one gender group.

In order to evaluate the individual predictive ability of the UK Biobank variables, we exhaustively fitted simple versions of our AutoPrognosis model for each of the 473 variables. For each such model, we use one distinct variable as an input and evaluate the resulting AUC-ROC. Because most variables are correlated with age and gender, we use the age variable as a second predictor for all models, and fit separate models for men and women. The AUC-ROC values of the resulting models are depicted in the scatter-plot in Fig 2.

As shown in Fig 2, variables related to smoking habits or exposure to tobacco smoke displayed the highest predictive ability. Self-reported health rating was predictive for both genders, but more predictive for women. Existence of long-standing illness was strongly predictive of CVD events for women, and less predictive for men. Variables extracted from the electrocardiogram (ECG) records possessed stronger predictive ability for men.

## Discussion

In this large prospective cohort study, we developed a ML model based on the AutoPrognosis framework for predicting CVD events in asymptomatic individuals. The model was built using data for more than 400,000 UK Biobank participants, with over 450 variables for each participant. Our study conveys several key messages. First, AutoPrognosis significantly improved the accuracy of CVD risk prediction compared to well-established scoring systems based on conventional risk factors and currently recommended by primary prevention guidelines (Framingham score). Second, AutoPrognosis was able to agnostically discover new predictors of CVD risk. Among the discovered predictors were non-laboratory variables that can be collected relatively easily via questionnaires, such as the individuals’ self-reported health ratings and usual walking pace. Third, AutoPrognosis uncovered complex interaction effects between different characteristics of an individual, which led to recognition of risk predictors that are specific to certain sub-populations for whom existing guidelines were providing unreliable predictions.

### When can ML help in prognostic modeling?

The abundance of a large number of informative variables in the UK Biobank (473 variables) guarantees an “information gain” that can be achieved by any data-driven model, including the standard Cox PH model, compared to the existing prediction algorithms that use only a limited number of conventional risk factors (e.g., Framingham score). The results in Table 2 show that, in addition to the information gain, AutoPrognosis also attained a “modeling gain” that allowed it to outperform the standard Cox PH model that uses all of the 473 variables. In general, the modeling gain achieved by AutoPrognosis would result from its ability to select among different models with various levels of complexity and numerical robustness in a completely data-driven fashion, without committing to any presupposition about the superiority of any given model. In our experiments, the Cox PH supplied with all of the 473 variables (without variable selection) provided a noticeably poor performance (i.e., an average AUC-ROC of 0.6). This is because the numerical solvers of the Cox PH model collapse when the data dimensionality is very large—this is why a variable selection pre-processing step was essential for fitting the Cox PH model. This implies that, even if the true underlying data model is perfectly linear, fitting standard linear models such as Cox PH or linear regression may not be sufficient for harnessing the information gain, since such models are not numerical robust in high-dimensional settings. AutoPrognosis solves this problem by selecting more robust models that better fit the high-dimensional data—in our experiments, these where tree-based models such as XGBoost and random forests. This observation shows that information gain and modeling gain are inherently entangled: to harness the information gain, we need to consider a more complex modeling space.

While the information gain appeared to be more significant than the modeling gain in our experiments, we note that even when provided with the same 7 core risk factors used by the Framingham score, AutoPrognosis was still able to offer a statistically significant AUC-ROC gain compared to the Framingham score and a Cox PH model that uses the same 7 variables. This shows that the modeling gain is not necessarily limited to settings where many predictors are available and numerical robustness, but is rather achievable whenever a small number of predictors display complex interactions.

Because not every ML model would necessarily improve over the Framingham score or the simple Cox PH model, our usage of the AutoPrognosis algorithm was essential for realizing the full benefits of ML modeling. As the results in Table 2 demonstrate, some ML models did not improve over the baseline Framingham score, whereas others provided modest improvements. This is because selection of the right ML model and careful tuning for the model’s hyper-parameters are two crucial steps for realizing the potential benefits of ML. AutoPrognosis automates those steps, which makes ML application easily accessible for mainstream clinical research. The importance of model selection and hyper-parameter optimization have been overlooked in previous clinical studies that applied ML in prognostic modeling [16–18]. Our study is unique in that, to the best of our knowledge, it is the first to carry out a comprehensive investigation of the performance of ML models in a large cohort with such an extensive number of predictors.

### Risk prediction with non-laboratory variables

Individuals in developed countries tend to seek out health information through online resources and web-based risk calculators [38]. In developing countries, where 80% of all world-wide CVD deaths occur [39], there are limited resources for risk assessment strategies that require laboratory testing [39, 40]. The results in Table 2 show that AutoPrognosis could potentially provide reliable risk predictions by using information from non-laboratory variables about the participants’ lifestyle and medical history. The most predictive non-laboratory variables included in our model were ages, gender, smoking status, usual walking pace, self-reported overall health rating, previous diagnoses of high blood pressure, income, Townsend index and parents’ ages at death. Inclusion of such variables in web-based risk calculators can help provide reasonably accurate risk predictions when obtaining laboratory variables is not viable.

One remarkable finding in Table 2 (and Fig 2) is that apart from the well-established age and gender risk factors, two other non-laboratory variables were found to be very predictive of the CVD outcomes; those are the “self-reported health rating”, and the “usual walking pace”. (Both variables were also found to be predictive of the overall mortality risk in a recent study on the UK Biobank [22].) Neither of the two variables is included in any of the existing risk prediction tools. Walking pace was equally predictive for men and women, but the self-reported health rating was more predictive for women and less for men. This may be explained by either gender-specific reporting bias or true clinical differences. Therefore, prediction tools that would include subjective non-laboratory variables, such as the self-reported health rating, should be carefully designed in such a way that self-reporting bias is reduced.

### Risk predictors specific to diabetic patients

Unlike the Framingham score, AutoPrognosis was able to maintain high predictive accuracy for participants diagnosed with diabetes at baseline (Table 4). This suggests that the AutoPrognosis model has learned diabetes-specific risk factors that were not previously captured by the existing prediction algorithms. By investigating the risk factor ranking within the diabetic subgroup (Table 5), we found that urinary microalbumin (measured in mg/L) is a very strong marker for increased CVD risk among individuals with diabetes. The dismissal of urinary microalbumin in existing risk scoring systems may explain their poor prognostic performance when validated in cohorts of diabetic patients [12, 13]. Our results indicate that predictions based on AutoPrognosis can provide better guidance for CVD preventive care in diabetic patients.

It is worth mentioning that the microalbumin in urine measures were available for only 125,406 participants in the overall cohort (29.6%). In a standard prognostic study, such a variable may get omitted from the analysis because of its high missingness rate. AutoPrognosis automatically recognized that this variable is relevant for diabetic patients, and hence did not omit it in its feature processing stage.

### Limitations

The main limitation of our study is the absence of the cholesterol biomarkers (total cholesterol, HDL cholesterol and LDL cholesterol) from the latest release of the UK Biobank data repository, which hindered direct comparisons with the QRISK2 scores currently recommended by the NICE guidelines. Furthermore, other blood-based biomarkers have been reported to be associated with CVD risk, but were also not yet released in the UK Biobank data repository, such as triglycerides [41], measures of glycemia [42], markers of inflammation [43], and and natriuretic peptides [44]. Inclusion of such predictors could improve the predictive accuracy of all models tested in this study, and could also alter the risk predictors’ ranking in Table 2, but is unlikely to change our conclusions on the usefulness of ML modeling in CVD risk prediction.

Another limitation of our study is that the UK Biobank cohort is ethnically homogeneous: 94% of the participants were of white ethnicity. Hence, assessment of the importance of ethnicity as a predictor of CVD events and the recognition of ethnicity-specific risk predictors was not possible in our study.

## Supporting information

S1 Table List of blood test measurements collected for the UK Biobank participants. (PDF)

S2 Table List of variables on the participants’ family history. (PDF)

S3 Table List of variables on the participants’ health and medical history. (PDF)

S4 Table List of variables on the participants’ dietary and nutritional information. (PDF)

S5 Table List of variables on the participants’ physical measures. (PDF)

S6 Table List of variables on the participants’ psychosocial status. (PDF)

S7 Table List of variables on the participants’ physical activity. (PDF)

S8 Table List of variables on the participants’ life style and environment. (PDF)

S9 Table List of variables on the participants’ sociodemographics. (PDF)

S1 Appendix Machine learning pipelines used by the AutoPrognosis model. (PDF)

S2 Appendix Explanation for the variables in Table 3. (PDF)
