# Supplementary material for: Cardiovascular disease risk prediction using automated machine learning: A prospective study of 423,604 UK Biobank participants
Source: PLoS One. 2019 May 15;14(5):e0213653. doi: 10.1371/journal.pone.0213653 (PMC6519796; doi:10.1371/journal.pone.0213653)
Supplement: S1 Table — (PDF) [file pone.0213653.s001.pdf]

White blood cell (leukocyte) count	Haematocrit percentage	Mean corpuscular Haemoglobin concentration
Red blood cell (erythrocyte) count	Haemoglobin concentration	Lymphocyte count
Red blood cell distribution width	Mean corpuscular volume	High light scatter reticulocyte percentage
Platelet (thrombocyte) volume	Platelet Crit	Neutrophill count
Platelet distribution width	Platelet count	Eosinophill count
Basophill count	Monocyte count	Nucleated red blood cell count
Lymphocyte percentage	Monocyte percentage	Neutrophill percentage
Eosinophill percentage	Basophill percentage	Reticulocyte percentage
Nucleated red blood cell percentage	Reticulocyte count	Mean reticulocyte volume

S1 Table List of blood test measurements collected for the UK Biobank participants.
