# Supplementary material for: Cardiovascular disease risk prediction using automated machine learning: A prospective study of 423,604 UK Biobank participants
Source: PLoS One. 2019 May 15;14(5):e0213653. doi: 10.1371/journal.pone.0213653 (PMC6519796; doi:10.1371/journal.pone.0213653)
Supplement: S2 Table — (PDF) [file pone.0213653.s002.pdf]

Respiratory	Gynecology/breast	Dermatology
Gastrointestinal/abdominal	Cerebrovascular disease	Obstetric problem
Renal/urology	Bowel problem	Hayfever, allergic rhinitis or eczema
Endocrine/diabetes	Other urological problem	Fracture head & neck
Neurology/eye/psychiatry	Neurology	Fracture upper limb & shoulder
Musculoskeletal/trauma	Infections	Other fractures
Hematology/dermatology	Hematology	Fracture pelvis & lower limb
Viral infection	Bacterial infection	Tropical infections
High blood pressure	Overall health rating	Diabetes Diagnosis
Blood clot in the leg (DVT)	Blood clot in the lung	Emphysema/chronic bronchitis
Asthma	Fracture	

S2 Table List of variables collected about a participant's mother/father/sibling clinical history.
