# Supplementary material for: Cardiovascular disease risk prediction using automated machine learning: A prospective study of 423,604 UK Biobank participants
Source: PLoS One. 2019 May 15;14(5):e0213653. doi: 10.1371/journal.pone.0213653 (PMC6519796; doi:10.1371/journal.pone.0213653)
Supplement: S3 Table — (PDF) [file pone.0213653.s003.pdf]

S3 Table Lists of variables on the participants' health and medical history.

Chest pain felt during physical activity	Hearing difficulty/problems
Chest pain felt outside physical activity	Falls in the last year
Neck/shoulder pain for 3+ months	Weight change compared with 1 year ago
Hip pain for 3+ months	Wheeze or whistling in the chest in last year
Chest pain or discomfort walking normally	Chest pain due to walking ceases when standing still
Back pain for 3+ months	Chest pain or discomfort
Stomach/abdominal pain for 3+ months	Chest pain or discomfort when walking uphill or hurrying
Knee pain for 3+ months	Headaches for 3+ months
Facial pains for 3+ months	Leg pain on walking

(a) List of symptoms presented by participants during their visit to the assessment center.

Number of Cancers	Histology of Cancer	Age at Cancer Diagnosis
Type of Cancer	Behavior of Cancer	

(b) List of variables related to the participant's cancer history.

Anti-hypertensive drugs	Other prescription medications
Lipid-lowering drugs	Breast cancer screening / mammogram
Number of Operations	Medication for cholesterol, blood pressure, diabetes
Number of Treatments	Medication for pain relief, constipation, heartburn
Bowel cancer screening	Vitamin and mineral supplements
Prostate specific antigen (PSA) test	Hearing aid user
Hormone-replacement therapy (HRT)	Pace-maker

(c) List of variables on the participant's treatment and screening history.

Respiratory	Gynecology/breast	Dermatology
Gastrointestinal/abdominal	Cerebrovascular disease	Obstetric problem
Renal/urology	Bowel problem	Hayfever, allergic rhinitis or eczema
Endocrine/diabetes	Other urological problem	Fracture head & neck
Neurology/eye/psychiatry	Neurology	Fracture upper limb & shoulder
Musculoskeletal/trauma	Infections	Other fractures
Hematology/dermatology	Hematology	Fracture pelvis & lower limb
Viral infection	Bacterial infection	Tropical infections
High blood pressure	Overall health rating	Diabetes Diagnosis
Blood clot in the leg (DVT)	Blood clot in the lung	Emphysema/chronic bronchitis
Asthma	Fracture	

(d) List of variables on the participant's history of non-cancer illnesses.
