# Supplementary material for: Cardiovascular disease risk prediction using automated machine learning: A prospective study of 423,604 UK Biobank participants
Source: PLoS One. 2019 May 15;14(5):e0213653. doi: 10.1371/journal.pone.0213653 (PMC6519796; doi:10.1371/journal.pone.0213653)
Supplement: S4 Table — (PDF) [file pone.0213653.s004.pdf]

Cooked vegetable intake	Salad and raw vegetable intake	Fresh fruit intake
Dried fruit intake	Oily fish intake	Non-oily fish intake
Processed meat intake	Poultry intake	Beef intake
Lamb intake	Pork intake	Cheese intake
Spreadable butter	Bread intake	Cereal intake
Salt added to food	Tea intake	Coffee intake
Water intake	Milk type used	Variation in diet
Alcohol intake	Average weekly red wine intake	Average weekly spirits intake
Average weekly beer plus cider intake	Average weekly champagne plus white wine intake	Average weekly fortified wine intake
Alcohol taken with meals	Soya Milk	Alcohol intake vs 10 years ago
Full cream milk	Semi-skimmed milk	White bread
Skimmed milk	Wholegrain bread	Brown bread
Decaffeinated coffee	Instant coffee	Ground coffee

S4 Table List of variables on the participants' dietary and nutritional information.
