# Supplementary material for: Cardiovascular disease risk prediction using automated machine learning: A prospective study of 423,604 UK Biobank participants
Source: PLoS One. 2019 May 15;14(5):e0213653. doi: 10.1371/journal.pone.0213653 (PMC6519796; doi:10.1371/journal.pone.0213653)
Supplement: S5 Table — (PDF) [file pone.0213653.s005.pdf]

Body Mass Index	Height	Weight	Pulse rate
Systolic blood pressure	Waist circumference	Hip circumference	T-score
Diastolic blood pressure	Body fat percentage	Leg fat-free mass R	Arm predicted mass R
Ankle spacing width	Impedance of leg R	Leg predicted mass R	Arm fat percentage L
Hand grip strength L	Impedance of leg L	Leg fat mass L	Arm fat mass L
Hand grip strength R	Impedance of arm R	Leg fat-free mass L	Arm fat-free mass L
Whole body fat-free mass	Impedance of arm L	Leg predicted mass L	Arm predicted mass L
Whole body fat mass	Leg fat percentage R	Arm fat percentage R	Trunk fat percentage
Whole body water mass	Leg fat percentage L	Arm fat mass R	Trunk fat mass
Basal metabolic rate	Leg fat mass R	Arm fat-free mass R	Trunk predicted mass

OCT measured (right)	Speed of sound through heel
OCT measured (left)	Heel quantitative ultrasound index (QUI)
Glasses worn/required (right)	Heel bone mineral density (BMD)
Glasses worn/required (left)	Contra-indications for spirometry
Birth weight	Heel Broadband ultrasound attenuation
Foot measured for bone density	Fractured heel

FEV1	FVC	PEF

S5 Table List of physical measures collected for participants.
