# Supplementary material for: Cardiovascular disease risk prediction using automated machine learning: A prospective study of 423,604 UK Biobank participants
Source: PLoS One. 2019 May 15;14(5):e0213653. doi: 10.1371/journal.pone.0213653 (PMC6519796; doi:10.1371/journal.pone.0213653)
Supplement: S6 Table — (PDF) [file pone.0213653.s006.pdf]

Mood swings	Miserableness
Irritability	Sensitivity/hurt feelings
Fed-up feelings	Nervous feelings
Worrier/anxious feelings	Tense/highly strung
Worry too long after embarrassment	Suffer from nerves
Loneliness and Isolation	Guilty feelings
Risk taking	Frequency of depressed mood in last 2 weeks
Freq. of unenthusiasm in last 2 weeks	Freq. of tenseness/restlessness in last 2 weeks
Seen doctor (GP) for nerves, anxiety, tension or depression	Frequency of tiredness/lethargy in last 2 weeks
Able to confide	Seen a psychiatrist for nerves, anxiety, tension or depression

S6 Table List of variables on the participants' psychosocial status.
