# Supplementary material for: Cardiovascular disease risk prediction using automated machine learning: A prospective study of 423,604 UK Biobank participants
Source: PLoS One. 2019 May 15;14(5):e0213653. doi: 10.1371/journal.pone.0213653 (PMC6519796; doi:10.1371/journal.pone.0213653)
Supplement: S7 Table — (PDF) [file pone.0213653.s007.pdf]

S7 Table List of variables on the participants' physical activity.

Sleep duration	Morning/evening person (chronotype)	Snoring
Getting up in morning	Sleeplessness / insomnia	Nap during day
Daytime sleeping (narcolepsy)		

(a) List of variables on the participants' sleeping patterns.

Duration of Walks	Slow pace walking	Job Involves Walking
Number of Days Walked	Brisk pace walking	Distance to Workplace
Unable to walk	Steady average pace walking	

(b) List of variables on the participants' walking patterns.

Days/Week of Moderate Physical Activity	Duration of Vigorous Activity	Frequency of Travelling
Days/Week of Vigorous Physical Activity	Duration of Moderate Activity	Job Involves Physical Work

(c) List of variables on the participants' work and activity patterns.

Frequency of strenuous sports	Duration of light DIY
Duration of strenuous sports	Frequency of light DIY

(d) List of variables on the participants' sports and do-it-yourself work practices.

Maximum workload during fitness test	Target heart rate achieved
Maximum heart rate during fitness test	Duration of fitness test
Able to walk or cycle unaided for 10 minutes	

(e) List of variables extracted from the fitness test conducted for participants at assessment centers.
