# Supplementary material for: Cardiovascular disease risk prediction using automated machine learning: A prospective study of 423,604 UK Biobank participants
Source: PLoS One. 2019 May 15;14(5):e0213653. doi: 10.1371/journal.pone.0213653 (PMC6519796; doi:10.1371/journal.pone.0213653)
Supplement: S8 Table — (PDF) [file pone.0213653.s008.pdf]

**S8 Table** Lists of variables on the participants' lifestyle and environment.

| Frequency of friend/family visits | Leisure/social activities |
|---|---|
| Time spend outdoors in summer | Time spent outdoors in winter |
| Time spent driving | Drive faster than motorway speed limit |

**(a)** List of variables on the participants' social interactions.

| Time spent watching television (TV) | Time spent using computer |
|---|---|
| Length of mobile phone use | Weekly usage of mobile phone in last 3 months |
| Hands-free device use in last 3 month | Plays computer games |
| Difference in mobile phone use compared to two years previously | |

**(b)** List of variables on the participants' interactions with technology.

| Current tobacco smoking | Smokers in household | Exposure to tobacco outside home |
|---|---|---|
| Past tobacco smoking | Exposure to tobacco at home | Maternal smoking around birth |

**(c)** List of variables on the participants' smoking habits.
