# Supplementary material for: Cardiovascular disease risk prediction using automated machine learning: A prospective study of 423,604 UK Biobank participants
Source: PLoS One. 2019 May 15;14(5):e0213653. doi: 10.1371/journal.pone.0213653 (PMC6519796; doi:10.1371/journal.pone.0213653)
Supplement: S9 Table — (PDF) [file pone.0213653.s009.pdf]

S9 Table Lists of variables on the participants' sociodemographic.

Qualifications	Adopted as a child	Number of full brothers
Maternal Status	Father/Mother still alive	Number of full sisters
Number of children fathered	Father/Mother age at death	Income

(a) List of variables on the participants' social background.

Townsend Deprivation Index	House or Bungalow
Flat or apartment	Caravan
Sheltered accommodation	Care home
Home rented/owned	Time in Current Address
Number in household	Number of vehicles
Length of Working Week	Time in Current Job
Transport type for commuting to job workplace	

(b) List of variables on the participants' general living conditions.
