# Supplementary material for: Cardiovascular disease risk prediction using automated machine learning: A prospective study of 423,604 UK Biobank participants
Source: PLoS One. 2019 May 15;14(5):e0213653. doi: 10.1371/journal.pone.0213653 (PMC6519796; doi:10.1371/journal.pone.0213653)
Supplement: S1 Appendix — (PDF) [file pone.0213653.s010.pdf]

S1 Appendix. Machine learning pipelines used by AutoPrognosis

AutoPrognosis created an ensemble of 200 machine learning pipelines, where each pipeline comprises a data imputation stage, a feature processing stage, a classification stage and a calibration stage. The final model is a weighted combination of the 200 pipelines. Because the model changes in every fold in the 10-fold cross-validation procedure, we list the 20 pipelines with the highest weights for an in-sample model that it fit to the entire cohort (423,604 participants).

Pipeline #	Imputation	Feature process.	Classification	Calibration	Weight
**1**	MissForest	No preprocess.	XGBoost	Sigmoid	0.345
**2**	Mean	No preprocess.	AdaBoost	Sigmoid	0.121
**3**	MissForest	No preprocess.	Gradient Boost.	Sigmoid	0.064
**4**	Mean	No preprocess.	Gradient Boost.	Sigmoid	0.064
**5**	MissForest	No preprocess.	Gradient Boost.	None	0.062
**6**	MissForest	PCA	XGBoost	Sigmoid	0.051
**7**	MICE	ICA	XGBoost	Sigmoid	0.023
**8**	EM	PCA	XGBoost	None	0.022
**9**	MICE	No preprocess.	XGBoost	Sigmoid	0.021
**10**	MissForest	Fast ICA	Random Forest	Sigmoid	0.021
**11**	Mean	Fast ICA	Logistic Reg.	Sigmoid	0.021
**12**	Mean	Fast ICA	Neural Net.	None	0.020
**13**	MICE	No preprocess.	XGBoost	Sigmoid	0.018
**14**	Mean	PCA	Neural Net.	Isotonic	0.018
**15**	MissForest	Fast ICA	Logistic Reg.	Sigmoid	0.017
**16**	Mean	PCA	Neural Net.	None	0.017
**17**	Mean	No preprocess.	XGBoost	None	0.017
**18**	MissForest	PCA	Neural Net.	Sigmoid	0.015
**19**	Mean	Fast ICA	Neural Net.	Sigmoid	0.013
**20**	MICE	PCA	Logistic Reg.	None	0.011
