# Supplementary material for: Cardiovascular disease risk prediction using automated machine learning: A prospective study of 423,604 UK Biobank participants
Source: PLoS One. 2019 May 15;14(5):e0213653. doi: 10.1371/journal.pone.0213653 (PMC6519796; doi:10.1371/journal.pone.0213653)
Supplement: S2 Appendix — (PDF) [file pone.0213653.s011.pdf]

S2 Appendix. Explanation for the variables in Table 3.

- **Usual walking pace:** Self-reported usual walking pace of the participant was self-defined as slow, steady/average or brisk.

- **Ankle spacing width:** Bone-densitometry of heel, the Ankle width is indicated by the spacing between measurement transducer pads on heel.

- **Self-reported health rating:** Participants' own assessment of their health defined as excellent, good, fair, poor.

- **Number of treatments:** Number of medications currently taken by the participant.

- **Leg predicted mass:** Estimated mass of the leg (measured in Kg) used to estimate body composition.

- **Basal metabolic rate:** The participant's resting metabolic rate as measured in the assessment center

- **Blood clot in the leg:** previous blood clots for the participants.

- **Duration of fitness test:** Duration of the fitness test that the participants went through in order to extract his/her ECG during exercise.
